# Antibiotic prescription for the prevention and treatment of postoperative complications after routine dental implant placement. A cross-sectional study performed in Spain

**DOI:** 10.4317/jced.54637

**Published:** 2018-03-01

**Authors:** Octavi Camps-Font, Marta Viaplana-Gutiérrez, Javier Mir-Mari, Rui Figueiredo, Cosme Gay-Escoda, Eduard Valmaseda-Castellón

**Affiliations:** 1 DDS, MS, Master of Oral Surgery and Implantology. Associate Lecturer in Oral Surgery and Lecturer on the Master of Oral Surgery and Implantology degree course, Faculty of Medicine and Health Sciences, University of Barcelona; 2 DDS. Specialty registrar, Master of Oral Surgery and Implantology degree course. Faculty of Medicine and Health Sciences, University of Barcelona; 3 DDS, MS, Master of Oral Surgery and Implantology. Lecturer on the Master of Oral Surgery and Implantology degree course, Faculty of Medicine and Health Sciences, University of Barcelona; 4 DDS, MS, PhD, Master of Oral Surgery and Implantology. Associate Lecturer in Oral Surgery and Lecturer on the Master of Oral Surgery and Implantology degree course, Faculty of Medicine and Health Sciences, University of Barcelona. Researcher at the IDIBELL institute; 5 MD, DDS, MS, PhD. Head of department and Professor of Oral and Maxillofacial Surgery, Faculty of Medicine and Health Sciences, University of Barcelona. Coordinating researcher at the IDIBELL Institute. Head of the Oral and Maxillofacial Surgery Department, Teknon Medical Center, Barcelona, Spain; 6 DDS, MS, PhD, Master of Oral Surgery and Implantology. Tenured Lecturer in Oral Surgery and Director of the Master of Oral Surgery and Implantology degree course, Faculty of Medicine and Health Sciences, University of Barcelona. Researcher at the IDIBELL institute

## Abstract

**Background:**

As there are no established guidelines for antibiotic prescription after dental implant placement a study was made to determine the current prescribing habits of several groups of practitioners regarding antibiotics to prevent and/or treat postoperative complications — early failures and infections — in relation to routine dental implant placement.

**Material and Methods:**

An electronic survey was sent to postgraduate students and professionals with experience in routine dental implant placement who practice in Spain. The questions asked were related to whether antibiotics were routinely prescribed either pre- or postoperatively to prevent and/or treat postoperative complications during routine dental implant placement, and, if so, what antibiotics, dosage, frequency, and duration were used. Descriptive and bivariate analyses of the data were performed.

**Results:**

Two hundred and forty-seven responses were obtained. Preventively, 17 respondents (6.9%) prescribed antibiotics only preoperatively (95% confidence interval (CI): 3.7 to 10.0%), 100 (40.5%) preferred to give them exclusively during the postoperative period (95%CI 34.4 to 46.6%) and 94 practitioners (38.1%) prescribed antibiotics both pre- and post-operatively (95%CI 32.0 to 44.1%). The most common preoperative regime was amoxicillin 2 g given orally 1 hour before the procedure (21.6%, n = 24) following amoxicillin 750 mg given orally 1 day prior to surgery (21.6%, n = 24). The most common routine postoperative regime was amoxicillin 750 mg given orally for 7 days (34.0%, n = 66). To treat postoperative infections during the osseointegration period, 233 respondents (93.2%) prescribed antibiotics (95%CI 91.4 to 97.2%). The most common regime used was amoxicillin and potassium clavulanate 875/125 mg, given orally for 7 days (51.9%, n = 121).

**Conclusions:**

There is no consensus among dental clinicians regarding antibiotic use during routine dental implant placement to prevent postoperative complications and/or early failures. Moreover, the most commonly-prescribed regimes differ from that recommend in the latest published studies.

** Key words:**Antibiotics, dental implants, oral implantology, complications, postoperative wound infection, early failure.

## Introduction

Dental implants are considered a safe and predictable therapeutic option with excellent outcomes both short- and long-term. However, as in any surgical procedure, complications can occur (1).

Early failures — before prosthetic loading — are an uncommon but important finding in implant dentistry, with incidences ranging from 1% to 2% according to most studies (2). Although their true pathogenesis remains unknown, it is believed that a certain number result from infections during the osseointegration period (3,4).

Three recently published meta-analyses have shown that the oral administration of 2 or 3 g of amoxicillin one hour prior to surgery significantly reduces the risk of dental implant failure (5-7). However, there is a lack of evidence concerning whether adjunctive use of postoperative antibiotics is beneficial and which antibiotic is the most effective (5). Moreover, these agents do not seem to reduce the incidence of postoperative infections significantly (5,6). In any case, as with any biomaterial infection, the treatment of such complications is quite complex and can persist until the implanted device is removed (8).

Despite the contradictory results and the absence of standardized guidelines, prophylactic antibiotics continue to be in widespread routine use with dental implant placement in order to prevent postoperative infections and early failures. In addition, very few papers focus on determining the treatment for patients afflicted with postoperative infections after dental implant placement.

Accordingly, the aims of the present study were to determine the current habits of several groups of practitioners regarding antibiotic prescriptions to prevent and/or treat postoperative complications — early failures and infections — in relation to routine dental implant placement.

## Material and Methods

-Study design

A cross-sectional internet-based survey of postgraduate students and professionals (i.e. general dentists, periodontists, oral surgeons and oral and maxillofacial surgeons) with experience in routine dental implant placement, practising in Spain, was made using software specifically designed for this purpose (SurveyMonkey, Palo Alto, CA, USA). An e-mail sent in June 2015 invited all the participants to answer the survey. The purpose of the study was clearly defined and participation in the study was optional. The resulting survey data were anonymous and confidential. In order to obtain a higher response rate, one follow-up e-mail was sent (9). All surveys completed before 31 July 2015 were included in the analysis.

-Questionnaire 

**Figure 1:**
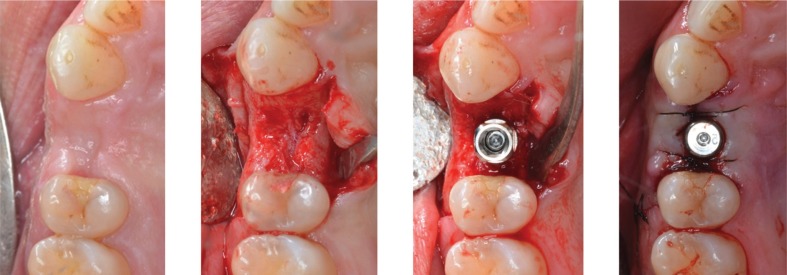
Hypothetical clinical case of routine dental implant placement.

A fifty-three question survey was developed, based on a hypothetical clinical case of a healthy 43 year-old patient, without antibiotic allergies or toxic habits, treated with routine dental implant placement (Fig. 1).

The first three questions addressed the participants’ demographics. Questions 4 to 35 related to whether antibiotics were routinely prescribed either pre- or postoperatively and, if so, what active ingredient, administration route, dosage and duration were used. Similarly, the last 18 questions concerned the antibiotic treatment — active ingredient, route of administration, dosage and duration — and prognosis for postoperative infections following dental implant surgery.

Because of the multiple-choice design, only 1 antibiotic regime could be selected in each of the sections. Moreover, due to the skip logic pattern applied, the path through the survey varied according to respondent’s answers. Hence, a total of 6 to 17 questions had to be answered.

-Statistical analysis

Statistical analysis was carried out with the STATA 14 statistical package (StataCorp, College Station, TX). A descriptive analysis was performed and the 95% confidence intervals (95%CI) were calculated for all prevalences. Parametric (Pearson’s χ2 test) and nonparametric tests (Fisher’s exact test) were used to compare the groups. The level of significance was set at a *p*-value of less than .05.

## Results

Two hundred and forty-seven responses (20.1%) were obtained from the 1227 surveys sent. The sample was heterogeneous regarding the clinical area of the professionals (*p* < 0.001) since the respondents were 86 (34.8%) oral surgeons, 71 (28.7%) general dentists, 36 (14.6%) postgraduate students, 34 (13.8%) periodontists and 20 (8.1%) maxillofacial surgeons. Sample heterogeneity (*p* < 0.001) was also noticed in relation to professional experience in placing dental implants, since 97 (39.3%) respondents had less than 5 years’ experience, 58 (23.5%) had 5 to 10 years, 59 (23.9%) 10 to 20 years and 33 (13.4%) more than 20 years. Most of the respondents mentioned an academic field linkage (67.6%, n = 167, *p* < 0.001).

Prevention of postoperative complications

Overall, 17 respondents (6.9%) prescribed routine antibiotics only preoperatively (95%CI: 3.7 to 10.0%) whereas 100 (40.5%) preferred to give them exclusively during the postoperative period (95%CI 34.4 to 46.6%), although 94 practitioners (38.1%) prescribed antibiotics both pre- and post-operatively during routine dental implant placement (95%CI 32.0 to 44.1%). No significant differences between those who prescribed antibiotics preoperatively, postoperatively or both pre- and post-operatively were found in relation to experience (*p*= 0.541; *p* = 0.600; *p* = 0.481) or to working in a University environment (*p* = 0.420; *p* = 0.294; *p* = 0.663). Conversely, regarding practice type, periodontists prescribed postoperative antibiotics significantly less often than the other groups (*p* = 0.002), but no differences were found between the percentages of those who prescribed preoperatively (*p* = 0.125) or both pre- and post-operatively (*p* = 0.146).

Of the 111 respondents who indicated that they prescribed preoperative antibiotics, the most common regimes used were amoxicillin 2 g given orally 1 hour before the procedure (21.6%, n = 24) and amoxicillin 750 mg given orally 1 day prior to surgery (21.6%, n = 24) ( Table 1). No significant differences between the active ingredients prescribed were found according to experience (*p* = 0.222, after excluding clindamycin and ‘other’ respondents) or working in a University environment (*p* = 0.147, after excluding clindamycin and ‘other’ respondents). Regarding practice type, maxillofacial surgeons prescribed amoxicillin and potassium clavulanate significantly more often than the other groups (*p* = 0.013, after excluding clindamycin and ‘other’ respondents) ( Table 2).

**Table 1:**
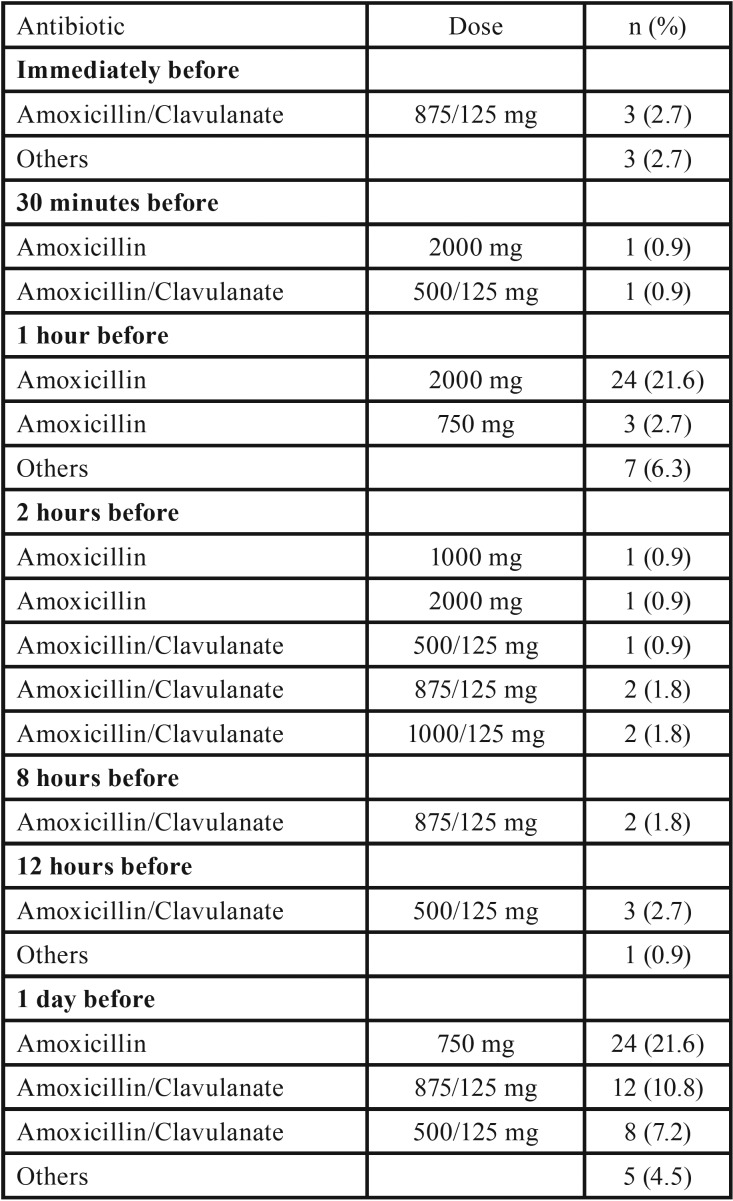
Preoperative prophylactic antibiotic regimes.

**Table 2:**
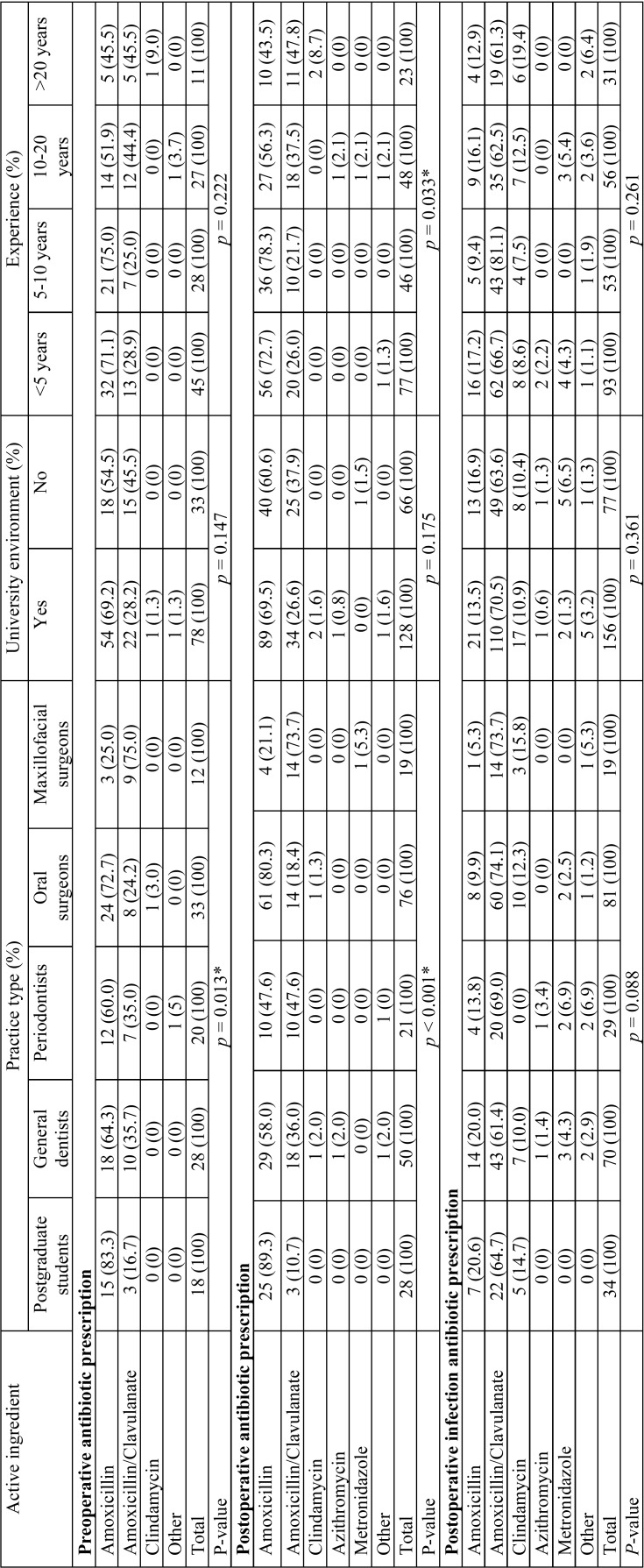
Results of bivariate analyses.

The most common regime among of the 194 respondents who reported postoperative antibiotic prescription was amoxicillin 750 mg given orally for 7 days (34.0%, n = 66), followed by amoxicillin and potassium clavulanate 875/125 mg given orally for 7 days (11.3%, n = 22) or amoxicillin 500 mg given orally for 7 days (11.3%, n = 22) ( Table 3). No significant differences in the active ingredient prescribed were found in relation to working in a University environment (*p* = 0.175, after excluding clindamycin, metronidazole, azithromycin and ‘other’ respondents). However, maxillofacial surgeons, periodontists and general dentists, as well as practitioners with over 20 years’ experience, prescribed amoxicillin and potassium clavulanate significantly more often than other groups (*p* < 0.001 and *p* = 0.033 respectively after excluding clindamycin, metronidazole, azithromycin and ‘other’ respondents) ( Table 2).

**Table 3:**
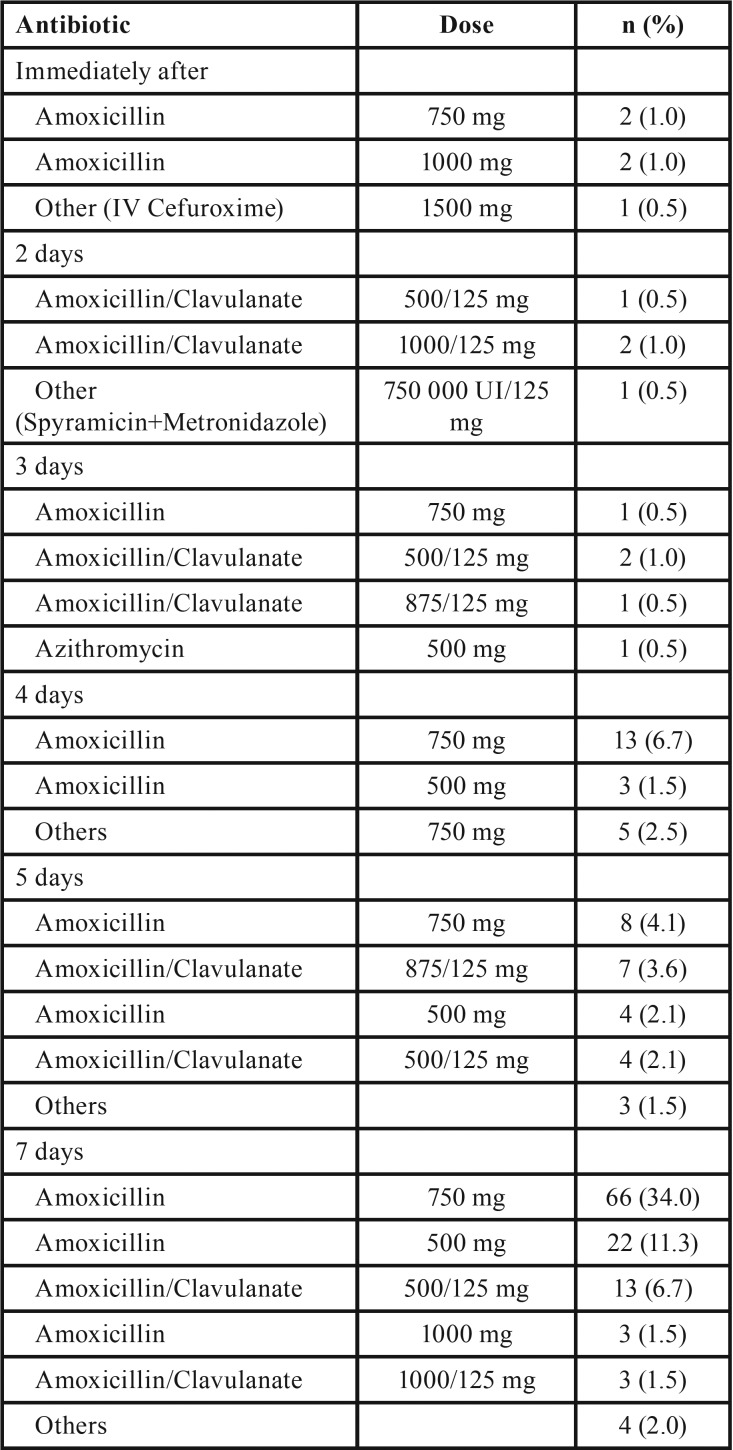
Postoperative prophylactic antibiotic regimes.

-Treatment of postoperative complications

Overall, 233 respondents (93.2%) prescribed antibiotics to treat postoperative infections during the osseointegration period (95%CI 91.4 to 97.2%). Among these, the regime most commonly used was amoxicillin and potassium clavulanate 875/125 mg given orally for 7 days (51.9%, n = 121). This was followed by amoxicillin 750 mg (9.0%, n = 21) and clindamycin 300 mg (8.6%, n = 20) given orally for 7 days ( Table 4). No significant differences in the active ingredient prescribed were found according to practice type (*p* = 0.088), working in a University environment (*p* = 0.175) or experience (*p* = 0.261) ( Table 2).

**Table 4:**
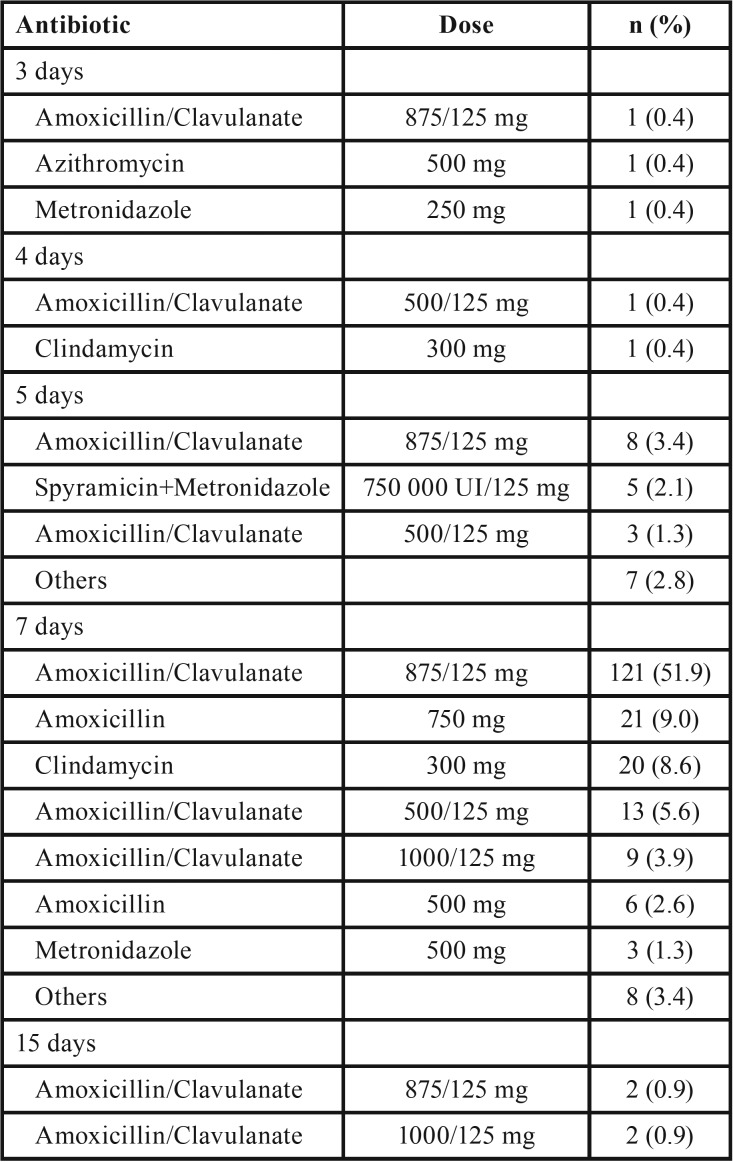
Antibiotic regimes for treating postoperative infections.

The respondents who considered that postoperative infections increase the risk of an early or late failure of the implants affected respectively numbered 232 (93.9%; 95%CI 90.9 to 96.9%) and 107 (43.3%; 95%CI 37.1 to 49.5%). No significant differences were found in perceived risk according to practice type (*p* = 0.346; *P* = 0.288), working in a University environment (*p* = 0.211; *P* = 0.196) or experience (*p* = 0.604; *P* = 0.120).

## Discussion

According to this study, there is no consensus regarding antibiotic use for preventing or reducing postoperative complications and/or early failures in routine dental implant placement ( Table 1, Table 2). Indeed, the vast majority of respondents did not prescribe these agents in accordance with the recommendations of published studies — 2 or 3 g of amoxicillin given orally 1 hour preoperatively (5-7) — as only 3.2% (8 out of 247) of them indicated such a protocol. Furthermore, a huge number of active ingredients, regimes and dosages was found.

The main limitation of the present study is the small number of questionnaires analysed due to the low response rate (20.1%). Moreover, it should be pointed out that those surveyed were contacted without using the database of any institution (e.g. professional associations or scientific societies), thereby facilitating the formation of heterogeneous groups and hindering the extrapolation of the results to the population of all Spanish professionals with experience in routine dental implant placement. However, in our opinion these results may help to understand the current antibiotic-prescribing situation in these professional communities. Another drawback is that questionnaires have to be simple, brief, quick and easy to read and complete. Consequently, important information that the clinician might consider relevant could be neglected.

When analysing antibiotic selection, most of the maxillofacial surgeons considered amoxicillin with clavulanate to be the first-line prophylactic drug in both the pre- and post-operative periods (75.0% and 73.7%, respectively). This finding is surprising, since very few authors advise using broad-spectrum antibiotics to prevent postoperative infections, especially in operations that have a low rate of complications like dental implant placement. These professionals clearly have different prescription criteria compared to the rest of the clinicians (*p* < 0.05). Nevertheless, such differences should be interpreted with caution due to the small number of maxillofacial surgeons (n = 19) included in the survey.

Despite the absence of any scientific evidence to support the use of antibiotics in the postoperative period after routine dental implant placement (5), it is surprising that most of the clinicians in this study (78.5%), with the exception of periodontists (*p* = 0.002), indicated that they prescribe postoperative regimes. In any case, it is important to stress that when antibiotics are prescribed to prevent infections, the duration of the treatment should be as short as possible (10). Hence, prolonged regimes may constitute an irrational use of such agents, increasing the likelihood of bacterial resistance and adverse drug reactions without significantly reducing the early failure or infection rates.

A recent study with a sample of 217 American oral and maxillofacial surgeons reported no consensus regarding antibiotic use during routine implant placement (11). Indeed, the proportions of subjects who indicated antibiotics preoperatively (51.6% vs 44.9%), postoperatively (71.4% vs 78.5%) or both pre- and post-operatively (34.0% vs 38.1%) were similar to the present results. Nevertheless, the American surgeons’ regimes tended to be shorter and at lower dosages, especially after the surgical procedure. Such differences might be due to both sociological and profession-related factors. It has to be taken into account that Spain is one of the European countries with the highest antibiotic consumption rates and, therefore, the highest percentages of bacterial resistance (12).

In the present study, amoxicillin and potassium clavulanate 875/125 mg given orally for 7 days was the regime selected by slightly over half the respondents (51.9%) to manage postoperative infections. This decision is probably based on the assumption that this combination theoretically covers the entire bacterial spectrum of odontogenic infections in Spain (13). However, it is known that biomaterial-based infections are extremely resistant to antibiotics and frequently persist until the implanted device is removed (8). Indeed, a recent study showed that in nearly three-quarters of cases (77.3%), most of them treated with amoxicillin and potassium clavulanate, an additional surgical procedure had to be performed in order to treat postoperative infections.

Several authors have reported considerably higher early implant failure rates when postoperative infections occur during the osseointegration period (4,14-16). Nevertheless, it is not known whether such a complication could jeopardize the long-term treatment outcome (17).

## Conclusions

There is no consensus among dental clinicians regarding the use of antibiotics for preventing postoperative complications and/or early failures in relation to routine dental implant placement. Moreover, the most common regimes prescribed differed in dosage and duration from that recommended in the latest published studies. There is an urgent need to unify criteria in order to prevent the appearance of antibiotic resistance and side effects.
